# Inhibition of androstenediol-dependent LNCaP tumour growth by 17*α*-ethynyl-5*α*-androstane-3*α*, 17*β*-diol (HE3235)

**DOI:** 10.1038/sj.bjc.6604987

**Published:** 2009-03-31

**Authors:** R Trauger, E Corey, D Bell, S White, A Garsd, D Stickney, C Reading, J Frincke

**Affiliations:** 1 Hollis-Eden Pharmaceuticals, San Diego, CA, USA; 2 Department of Urology, University of Washington, Seattle WA, USA; 3 Merck & Co Inc., North Wales, PA, USA

**Keywords:** prostate cancer, LNcaP, apoptosis

## Abstract

Androst-5-ene-3*β*, 17*β*-diol (AED) is an adrenal hormone that has been reported to sustain prostate cancer growth after androgen deprivation therapy (ADT). LNCaP cells express a mutated androgen receptor that confers the ability to respond not only to androgen but also to oestrogen and adrenal hormones such as AED, and thus provide a cell line useful for identifying compounds capable of inhibiting AED-stimulated cell growth. We sought to determine whether structurally related steroids could inhibit AED-stimulated LNCaP cell growth *in vitro* and tumour growth *in vivo*. We report here the identification of a novel androstane steroid, HE3235 (17*α*-ethynyl-5*α*-androstan-3*α*, 17*β*-diol), with significant inhibitory activity for AED-stimulated LNCaP proliferation. This inhibitory activity is accompanied by an increase in the number of apoptotic cells. Animal studies have confirmed the cytoreductive activity of HE3235 on LNCaP tumours. The results suggest that this compound may be of clinical use in castration-resistant prostate cancer.

A considerable amount of research has been directed towards understanding factors that support the growth and expansion of prostate cancer during and after anti-androgen treatment. The observation that androgen deprivation results in increase in AR expression (Scher *et al*, 2004) highlights the importance of AR in this stage of disease and suggests that other ligands may be capable of binding and activating AR to drive tumour cell growth. It is well documented that mutations in the AR allow castration-resistant prostate cancer cells to utilise a broad spectrum of steroids, whose levels are not affected by anti-androgen therapy (Taplin *et al*, 1995). These include oestrogen, progesterone, glucocorticoid, and the adrenal hormone androst-5-ene-3*β*, 17*β*-diol (AED), a multi-potent hormone that is an androgen and oestrogen precursor, with the ability to directly transactivate AR and ER in prostate cancer cells (Miyamoto *et al*, 1998; Maggiolini *et al*, 2004).

Because AED's central role both as a precursor of DHT and a direct activator of the androgen receptor, we initiated studies to identify an inhibitor of AED signalling in hormonally sensitive tumour cells from a proprietary chemical library based on the structure of AED. Through the course of this work, compounds were identified that inhibited the proliferation of LNCaP cells stimulated by AED *in vitro*. Subsequent work to identify the most suitable pharmaceutical from this series of compounds resulted in the identification of HE3235 (17*α*-ethynyl-5*α*-androstan-3*α*, 17*β*-diol). Here, we present results showing that HE3235 blocks the growth of LNCaP cells by inducing apoptosis. Tumour studies have confirmed the *in vivo* activity of HE3235 against AED-stimulated LNCaP tumour cells.

## Materials and methods

### Synthesis of HE3235

HE3235 was synthesised by reacting trimethylsilylazine with androsterone (DGP, Shanghai, China) in refluxing acetonitrile using saccharin catalyst. The trimethylsilyl-protected intermediate was subsequently reacted with ethynyltrimethylsilane activated with *n*-butyl lithium in tetrahydrofuran. The crude product was then recovered by slow addition of water to quench the reaction and filtration of the crude precipitate. The reaction product was purified to >99% pure HE3235 with a 67% of overall yield.

### LNCaP proliferation assay

LNCaP-FGC, PC3, and DU145 cells were obtained from the American Type Tissue Collection (ATTC, Manassas, VA, USA). To identify compounds with anti-proliferative activity for LNCaP, cells were seeded in poly-D-lysine coated 96-well plates at 3.75 × 10^4^ per ml in a total volume of 200 *μ*l of RPMI per 10% foetal bovine serum (FBS). After incubation overnight at 37°C, 5% CO_2_, the media was removed and the cells were washed once with 150 *μ*l per well of phenol red-free RPMI containing 10% charcoal per dextran-treated FBS (CS-RPMI) containing 10 nM AED. The appropriate dilution of each test compound was added and the cells were incubated for 5 days at 37°C, 5% CO_2_. For IC_50_ determination, a dilution sequence of compound was analysed and the data were modelled using a nonlinear regression, sigmoidal dose response, variable slope equation. The curve fit was constrained with a bottom of 0 and a top of 100. IC_50_ values were generated by GraphPad Prism 4. Cell proliferation was measured on day 5 using the CyQuant assay (Invitrogen, Carlsbad, CA, USA). The affect of HE3235 on cell growth of LNCaP, PC3, and Du145 cells was assayed by plating 3 × 10^5^ cells in 5% CS-RPMI supplemented with 10 nM AED. After 4 days, cells were manually counted with a haemocytometer.

### Apoptosis assay

LNCaP cells (5 × 10^5^) were seeded in phenol red-free RPMI with 5% charcoal-stripped serum (CSS) in 6-well plates and allowed to adhere overnight, then cultured in CSS supplemented with 10 nM AED with or without 50 nM HE3235 for 4 days. At the end of the incubation period, floating and adherent cells were harvested for analysis. Apoptosis was measured using the Annexin V-FITC Apoptosis Detection Kit (Calbiochem, La Jolla, CA, USA) according to the instructions of the manufacturer to detect apoptosis. PI and Annexin positive cells were considered apoptotic.

### Androgen-mediated transcription assays

The ARE-luciferase reporter construct (probasin promoter containing ARE) was provided by Dr Plymate (University of Washington, Seattle, WA, USA), and the 5.8 kb PSA promoter-luciferase plasmid was provided by Dr Kim (Fred Hutchinson Cancer Research Center, Seattle, WA, USA). An Amaxa Nucleofector device was used for transfection of LNCaP cells using solution V and program T-27 according to the manufacturer's directions (Amaxa Biosystems Inc., Gaithersburg, MD, USA). LNCaP cells (1 × 10^6^) were transfected with ARE-reporter plasmid or PSA-promoter reporter plasmid (1 *μ*g). The hTK renilla-luciferase plasmid (2 ng) was transfected under the same conditions to enable normalisation of transfection efficiencies. Cells were seeded in 24-well plates in phenol red-free RPMI with 5% CSS to allow adherence overnight, and then DHT, E2, or AED were added (1 nM or 10 nM final concentration) with or without 50 nM HE3235 in DMSO. Cells were incubated for ∼48 h, lysed and subjected to a dual-luciferase assay according to manufacturer's recommendations (Promega, Madison, WI, USA) using Tecan Genios plus luminometer. The luciferace activity was measured and signal normalised to transfection efficiencies based on TK transfection.

### AED-stimulated LNCaP tumour model

All animal studies were performed within the established IACUC guidelines. Castrated SCID mice (6 week old) were obtained from Jackson Laboratories (Bar Harbor, ME, USA). After 4 days of acclimation, the mice were implanted with AED pellets (5 mg, 60-day time-release, IRA, Sarasota, FL, USA). After 3 days, all mice were injected subcutaneously in the right flank with 100 *μ*l of 7.5 × 10^6^ LNCaP tumour cells in phenol red-free RPMI mixed 1 : 1 with Matrigel (BD, Franklin Lakes, NJ, USA). Tumour volumes were measured weekly and calculated as *a*^2^ × *b*/2 with *a* being the width and *b* the length of the tumour in mm (reported as mm^3^). HE3235 was prepared for injection by dilution in Captisol (CyDex, Lenexa, KS, USA).

To test the effect of dose on tumour incidence, a total of 48 castrated male SCID mice were implanted with LNCaP tumour cells as described above, and the mice were randomized into four groups of 12 animals each (vehicle control, 4 mg per mouse per day, 1 mg per mouse per day, and 0.4 mg per mouse per day). Administration of HE3235 (200 *μ*l intraperitoneally) or vehicle began 24 h after tumour inoculation. All animals were dosed daily for 28 consecutive days.

To test the effect of HE3235 on established LNCaP tumours, 36 castrated SCID mice received AED pellets and were implanted with LNCaP tumour cells and monitored as described above. Once the tumours reached 15–25 mm^3^, the mice were paired by tumour volume, and each mouse in a pair was assigned to either the vehicle or 4 mg per mouse per day HE3235 group. Animals were dosed IP with 200 *μ*l HE3235 or vehicle once a day for 21 days.

### Statistical analyses

The incidence of a measurable tumour was estimated as the relative frequency of mice having a measurable tumour at least once, any time in the experiment. The significance of the difference in incidence from vehicle was tested by means of Fisher's exact test, adjusted for multiplicity of comparisons (Westfall *et al*, 1999). Dose response in the proportion of mice with tumour was tested for significance through exact Cochran–Armitage test (StatXact 2004). Time to first measurable tumour volume is analysed through Kaplan–Meier product-limit estimates, with the exact logrank test applied to test for the significance of the differences (Cantor, 1997). Tumour volumes and tumour growth rates are analysed non-parametrically through exact Wilcoxon–Mann–Whitney test. Resulting *P*-values are corrected for multiplicity of comparisons (Westfall *et al*, 1999).

Reduction of tumour volume refers to a reduction persisting to the end of the study. Fisher's exact test was applied to test the significance of the difference in the incidence of such reduction relative to vehicle.

Time to first measurable tumour volume is analysed through Kaplan–Meier product-limit estimates, with the exact logrank test applied to test for the significance of the difference. Reduction of tumour volume is defined as a reduction in volume of at least 20% of the baseline volume, persisting to the end of the study. To detect the difference between active and control group, Fisher's exact test and exact 95% CI for the difference are applied. A tumour of non-measurable volume is a tumour that, with the methodology at hand, measures 0 to the end of the study. The growth rate of a tumour is also analysed through the mixed model.

## Results

### HE3235 blocks proliferation of LNCaP *in vitro*

**Figure 1:**
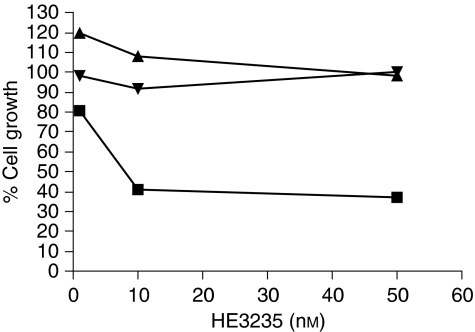
LNCaP (▪), PC3 (▴), and DU145 (⧫) cells were incubated with HE3235 at 1 nM, 10 nM, and 50 nM and assayed in duplicate for inhibition of cell growth relative to vehicle as described in Materials and Methods.

Through titration experiments, a concentration of 10 nM AED was found to provide maximal stimulation of LNCaP, and no proliferation of LNCaP cells was observed without the addition of AED (data not shown). Compounds ranked for potency by titration to calculate the IC_50_ of the compound. One compound, HE3235, inhibited LNCaP proliferation in the low nanomolar range (6 nM). This compound reduced LNCaP levels by 60% but had no inhibitory effect on the AR-negative PC3 and DU145 cells (Figure 1).

### LNCaP tumour model

**Figure 2:**
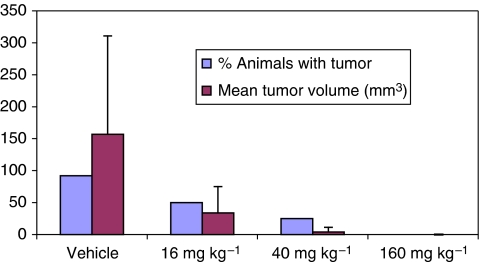
Dose-dependent effect of HE3235 on AED-stimulated LNCaP tumour incidence and tumour volume in SCID mice. Grey bars represent the percentage of animals in each dose group with tumour. Dark bars represent the average tumour volume (±s.d.).

Treatment with HE3235 resulted in a significant reduction in tumour incidence (compared with vehicle) in the two highest dose groups, (40 mg kg^−1^, *P*=0.006, *n*=11; 160 mg kg^−1^, *P*<0.001, *n*=12), with decreases in tumour volume apparent in all three dose groups (Figure 2). The mean tumour volume in the animals that developed tumours were also significantly affected, 157 mm^3^ (±153) (vehicle) *versus* 0 (*P*<0.001), 4 (±7) (*P*<0.001), 34 (±41) (*P*=0.004) mm^3^ in the HE3235 treated animals (descending dose). There was also a statistically significant delay in the time to a measurable tumour volume in all treated groups (*P*<0.01) relative to the vehicle control group.

**Figure 3:**
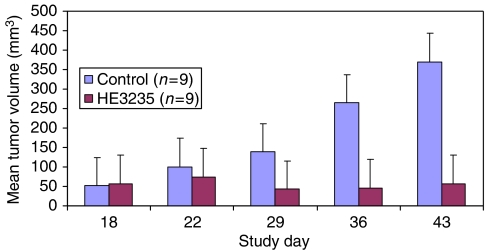
Effect of LNCaP of HE3235 on existing AED-stimulated tumours in SCID mice. LNCaP tumours were established according to Materials and Methods. Once the tumours reached at least 50 mm^3^, mice were separated to treatment (*n*=9) and vehicle control (*n*=9) groups and dosed as described in Materials and Methods.

Anti-tumor activity was also observed in animals with established tumours. As seen in Figure 3, vehicle-treated animals showed a progressive increase in tumour volume over the course of the study. In contrast, HE3235 treatment significantly blocked the growth of tumours (*P*<0.001). Significant differences in tumour volumes between the control and treated groups were observed by the first week and were maintained through the course of this study (*P*<0.001). A significantly greater percentage of mice in the treatment group reduced their tumour volumes by 20% or more (*P*<0.03), whereas no tumour reduction was seen in the vehicle group. Furthermore, tumours in two animals receiving HE3235 became non-measurable by day 15 of the study.

### HE3235 activates the mutant AR in LNCaP and stimulates PSA secretion

**Figure 4:**
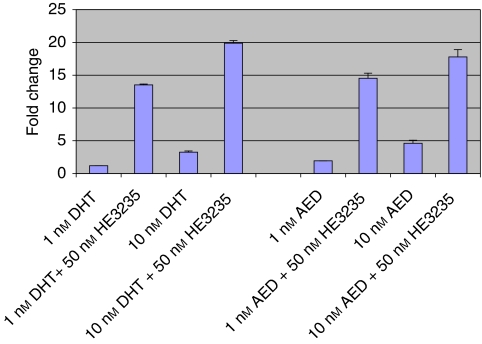
HE3235 activation of LNCaP AR. LNCaP cells were treated with 1 or 10 nM DHT or AED, charcoal-stripped serum (CSS). The fold change above background is shown on the *y* axis.

To determine whether HE3235 inhibited the activation of the LNCaP AR, LNCaP cells were transfected with an ARE and incubated with HE3235 with or without DHT and AED. As can be seen in Figure 4, HE3235 did not inhibit the activation of the LNCaP AR when the cells were stimulated with either DHT or AED. Instead, HE3235 activated AR in this setting. Consistent with this finding, an increase in amount of PSA secretion per cell was observed after incubation with HE3235. PSA rose from 8.5 ng per ml per 10^5^ cells to 175 ng per ml per 10^5^ cells by day 4 in LNCaP cells cultures incubated with 50 nM HE3235.

### HE3235 induces apoptosis in LNCaP

**Figure 5:**
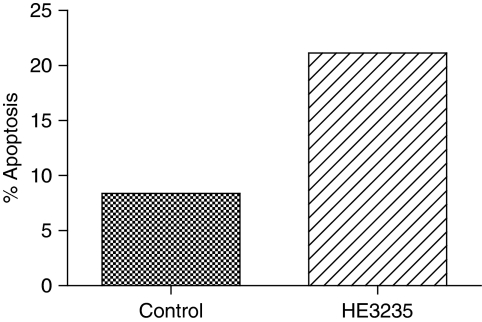
HE3235 induced apoptosis in LNCaP. The percentage increase in the number of apoptotic cells after 4 days of incubation with 50 nM HE3235 relative to the vehicle control is represented on the *y* axis.

Based on the observations above, the possibility that HE3235 was affecting cell viability in LNCaP cells was examined. Figure 5 shows a modest increase (8–21%) in the percentage of apoptotic LNCaP cells after 4 days of culture with HE3235, suggesting that HE3235 is cytotoxic for LNCaP cells.

## Discussion

The human prostate cancer cell-line LNCaP expresses a mutant androgen receptor that allows it to respond to a wide variety of hormones, including the adrenal hormone AED (Miyamoto *et al*, 1998; Chang *et al*, 1999; Mizokami *et al*, 2004; He and Falany, 2007). Because of its potential role in sustaining prostate cancer cell growth after androgen deprivation therapy (ADT), we set out to determine whether a compound capable of inhibiting AED-stimulated LNCaP cell proliferation could be identified from a proprietary steroid library that was developed around the structure of AED. As described here, this library yielded a novel androstane structure, HE3235, which blocked the proliferation of AED-stimulated LNCaP cells. This compound had no inhibitory effect on the AR-negative PC3 and DU145 cells (data not shown), suggesting that expression of the AR was required for inhibitory activity. When administered to animals at the time of LNCaP tumour challenge, HE3235 delayed the onset of the tumours and reduced tumour volumes in a dose-dependent manner, with a 100% inhibition of LNCaP tumours in the highest dose group. When treatment was applied to mice with existing tumours, a reduction in tumour volume was observed by the end of the first week of treatment, and tumour growth continued to be depressed throughout the course of the study. In addition, two out of nine animals treated with HE3235, in this study, had no detectable tumour volume by the end of the observation period. These results show that HE3235 had a cytoreductive effect on tumour volume.

Although the precise mechanism of action of HE3235 still remains to be elucidated, based on the *in vitro* and *in vivo* results presented here it would appear that HE3235 is not solely blocking the AED-stimulation of LNCaP tumours, rather, this anti-neoplastic activity seems to be through the induction of apoptosis. The exact mechanism through which HE3235 is inducing apoptosis is unclear. The observation that HE3235 did not inhibit the proliferation of PC3 and DU145 cells would suggest that the presence of the AR is required for its activity.

It is well established that DHT can cause a G_1_ arrest, but not cell death of LNCaP cells at concentrations above 10^−10^ nM (Tsihlias *et al*, 2000). Based on the results of the ARE studies, an experiment was conducted to determine whether HE3235 had a similar cytostatic effect on LNCaP. We also sought to determine whether, unlike DHT, exposure to HE3235 caused apoptosis in LNCaP cells. The results of these experiments showed that HE3235 does induce a modest increase in apoptosis. This finding would be consistent with the reduction in tumour volumes seen in mice treated with HE325. These effects are reminiscent of other agents reported to block the proliferation of prostate cancer cells *in vitro* and *in vivo*, most notable the histone deactylase inhibitors (HDACi). Using the LNCaP tumour model, sodium butyrate caused G_1_ cell-cycle arrest, inhibited LNCaP cell growth and increased PSA gene expression (Kruh, 1982; Gleave *et al*, 1998). Similar findings with respect to inhibition of cell proliferation and PSA secretion have been reported for vitamin D_3_ (Esquenet *et al*, 1996), calcitriol (Bauer *et al*, 2003), activin A, (Fujii *et al*, 2004) and phenylacetate (Walls *et al*, 1996). Future work will be directed towards further understanding whether the molecular mechanism of action of HE3235 is associated with an HDACi activity.

For many decades, androgen suppression has been the central theme for treating recurrent prostate cancer, yet virtually no patients are cured by this therapy. Prostate cancer progresses from androgen-dependent (AD) tumours that respond favourably to androgen ablation, to castration-resistant metastatic tumours that are invariably fatal. Pharmacological methods of lowering androgen levels in patients include the use of hormones that decrease androgen production, agents that block androgen association with the androgen receptor, and inhibitors of enzymes involved in androgen synthesis. Nonetheless, androgen receptor signalling remains active in castration-resistant prostate cancer despite these attempts at suppression. There are currently very few treatment options for men with castration-resistant prostate cancer. Androgen deprivation therapy induces a remission in 80–90% of men with advanced prostate cancer, and can increase median progression-free survival by 21 months (Hellerstedt and Pienta, 2002; Suzuki *et al*, 2007). This approach eventually fails to stop the disease progression because prostate tumour cells evolve to a castration-resistant state, in which tumours develop the ability to grow in castrate levels of T and DHT at least in part owing to the presence of AED. As described here, HE3235 represents a therapy that blocks the growth of AED-stimulated tumour cell growth, and thus, could be considered as a candidate for the treatment of castration-resistant prostate cancer.
